# The effects of buthionine sulfoximine treatment on diaphragm contractility and SERCA pump function in adult and middle aged rats

**DOI:** 10.14814/phy2.12547

**Published:** 2015-09-14

**Authors:** Ian C Smith, Chris Vigna, Andrew S Levy, Steven G Denniss, James W E Rush, A Russell Tupling

**Affiliations:** Department of Kinesiology, University of WaterlooWaterloo, Ontario, Canada

**Keywords:** Cellular redox, glutathione, oxidative stress, skeletal muscle

## Abstract

This study examined the effects of 10 days of buthionine sulfoximine (BSO) treatment on in vitro contractility and sarcoplasmic reticulum calcium pump (SERCA) expression and function in adult (AD; 6–8 months old) and middle aged (MA; 14–17 months old) rat diaphragm in both the basal state and following fatiguing stimulation. BSO treatment reduced the cellular concentrations of free glutathione (GSH) by >95% and oxidized glutathione (GSSG) by >80% in both age cohorts. GSH content in AD Control diaphragm was 32% higher (*P *<* *0.01) than in MA Control, with no differences in GSSG. The ratio of GSH:GSSG, an indicator of cellular oxidative state, was 34.6 ± 7.4 in MA Control, 52.5 ± 10.1 in AD Control, 10.6 ± 1.7 in MA BSO, and 9.5 ± 1.1 in AD BSO (BSO vs. Control, *P *<* *0.05). Several findings suggest that the effects of BSO treatment are age dependent. AD BSO diaphragm had 26% higher twitch and 28% higher tetanic force (both *P *<* *0.05) than AD Controls, whereas no significant difference existed between the two MA groups. In contrast to our previous work on BSO-treated AD rats, BSO treatment did not influence maximal SERCA ATPase activity in MA rat diaphragm, nor did SERCA2a expression increase in BSO-treated MA diaphragm. Biotinylated iodoacetamide binding to SERCA1a, a specific marker of free cysteine residues, was reduced by 35% (*P *<* *0.05) in AD Control diaphragm following fatiguing stimulation, but was not reduced in any other group. Collectively, these results suggest an important role for redox regulation in both contractility and SERCA function which is influenced by aging.

## Introduction

It is well established that a basal level of reactive oxidants is necessary for normal muscle function (Reid et al. 1993; Reid, 2001a; Smith and Reid 2006). Reid (Reid, 2001b) has developed a biphasic model describing the inotropic response of muscle to changes in the redox state, that is, the balance between the oxidizing species and the antioxidant species. In this model, the optimum point for force production occurs in the presence of low concentrations of reactive oxygen or nitrogen species (ROS, RNS), where both increases and decreases in ROS concentration decrease contraction force. In the basal state, the muscle typically has lower than optimum ROS concentration so that an increase in ROS production, such as that induced by exercise or exposure to low doses of exogenous ROS, will enhance muscle force. Exposure to higher doses of ROS such as those occurring during fatiguing exercise or high concentrations of exogenous ROS, will have a negative inotropic effect, which may be reversed or prevented by antioxidant exposure.

Glutathione is an important nonenzymatic antioxidant produced endogenously in muscle cells (Pastore and Piemonte 2012). It is an abundant source of free thiol groups which serve as electron donors in the glutathione peroxidase (GPX) reaction. GPX catalyzes the conversion of peroxides and H_2_O_2_ and reduced glutathione (GSH) to oxidized glutathione (GSSG) thereby reducing oxidative damage. Cellular GSH can be depleted by buthionine sulfoximine (BSO) which irreversibly inhibits *γ*-glutamylcysteine synthetase, a regulator of de novo GSH synthesis (Akai et al. 2007). When BSO was administered to the drinking water of adult rats, in addition to depleting GSH, the GSH:GSSG ratio was reduced in the diaphragm muscle (Morales et al. 1994; Tupling et al. 2007), representing an oxidative shift in the cellular redox state. Consistent with Reid’s model (Reid, 2001b), the BSO treatment resulted in increased force in isolated diaphragm strips and increased susceptibility to fatigue following repetitive stimulation (Morales et al. 1994; Tupling et al. 2007). We have additionally demonstrated that BSO treatment affects Ca^2+^ handling properties, with higher maximum rates of ATP hydrolysis and Ca^2+^ uptake by the sarco(endo)plasmic reticulum Ca^2+^-ATPase (SERCA) in freshly homogenized diaphragm (Tupling et al. 2007). However, following repetitive contractile activity, while untreated diaphragm exhibited an increase in ATPase activity and Ca^2+^ uptake by SERCA, there were no such increases in the BSO-treated diaphragm, indicating that ROS may play a role in contraction-induced activation of SERCA (Tupling et al. 2007). In addition, expression of the SERCA2a, but not SERCA1a, isoform was elevated in the rats treated with BSO (Tupling et al. 2007).

Oxidative stress has been suggested to be a major consequence of aging (Harman 1956), where oxidative damage accumulates leading to progressive reductions in cellular function, particularly in metabolically active, postmitotic tissues such as muscles and nerves (Barreiro 2014). This oxidative stress, which may result from increased ROS or RNS production, reduced antioxidant activity, or altered sensitivity of proteins to oxidative modifications, results in oxidative shifts in the cellular redox state (reviewed in Reid and Durham 2002). For example, the tibialis anterior of aged rats has been reported to exhibit a lower basal GSH:GSSG ratio than young controls (Ryan et al. 2008). In this same study, three weekly sessions of 80 unilateral concentric/eccentric contraction cycles for 4.5 weeks further lowered the GSH:GSSG ratio in the tibialis anterior of aged, but not young rats, suggesting an impaired ability to cope with the increased oxidative demands imposed by the loading protocol. Several studies have noted increased oxidation and nitration of SERCA under basal conditions in aged animals (Viner et al. 1997, 1999; Fugere et al. 2006; Sharov et al. 2006; Qin et al. 2013), which may lower the sensitivity of Ca^2+^-handling in skeletal muscle to changes in the redox state, however, there is limited information regarding the effects of redox state on Ca^2+^ handling in middle aged (MA) animals.

### Purpose and hypotheses

This study examined how the changes in diaphragm contractility and SERCA function associated with BSO treatment are influenced by aging in both the basal and fatigued states. It was expected that the cellular environment would become more oxidized in MA diaphragm, and that further oxidative shifts would occur following BSO-induced glutathione depletion. Based on these expectations and Reid’s model, it was anticipated that MA diaphragm would have an impaired ability to respond to the changes in the redox environment caused both by BSO treatment and by fatiguing stimulation compared with our previous study in younger animals. Specifically, we hypothesized that SERCA expression, function, and oxidation and diaphragm contractility would have lower responses to the oxidative shift imposed by BSO treatment in the MA animals. The methods used in this study have largely been chosen to facilitate comparison to our previous study on BSO treatment in adult rat diaphragm (Tupling et al. 2007).

## Methods

### Animal description and care

All experiments and protocols were approved by the University of Waterloo Animal Care Committee and were in accordance with the guidelines of the Canadian Council on Animal Care. Male Sprague Dawley rats were housed in a climate controlled facility on a reverse 12 h light–dark cycle. All rats were fed a diet of 22/5 rodent diet lab chow (Harlan). At the time of experimentation, rats were either 6–8 months (Adult; AD) or 14–17 months (MA) of age. The normal lifespan of the Sprague Dawley rat is ˜24 months (Masoro 1980), and the mortality rate in the MA group was 9%. Prior to experimentation, rats from the AD and MA cohorts were randomly assigned to either the control group (CTL) or the BSO-treated group, and either continued receiving normal tap water or received tap water containing 30 mmol/L BSO for 10 consecutive days, respectively. This dosage has been used previously by our group (Ford et al. 2006; Tupling et al. 2007), and others (Vaziri et al. 2000; Zhou et al. 2002; Bayorh et al. 2003) and is effective in lowering glutathione content with low toxicity over a 1- to 2-week period. As BSO is water soluble, no solubilizing vehicle was required. The mean ages of the resulting groups at time of sacrifice were 210 ± 6 days for AD Control, 459 ± 6 days for MA Control, 232 ± 5 days for AD BSO, and 451 ± 5 days for MA BSO. Following assignment to one of the groups, animals were housed individually in order to approximate the dosage of BSO each rat received. The animals used in this study were also used to study the effects of GSH depletion in vivo on carotid artery function (Denniss et al. 2011) and apoptotic signaling in skeletal muscle (Dam et al. 2012).

### Glutathione detection

The effects of BSO treatment on costal diaphragm glutathione content were assessed as described previously (Tupling et al. 2007). Briefly, homogenates were prepared from fresh sections of costal diaphragm and glutathione was assayed using high-performance liquid chromatography as described in Reed et al. (Reed et al. 1980). Homogenates were prepared on ice with a 1:10 dilution in homogenizing media (2 mmol/L phenanthroline in 7% perchloric acid) using handheld glass homogenizers. Following centrifugation of samples at 150 × *g* for 10 min at 4°C, 250-*μ*L aliquots of supernatant were removed and treated with 10 *μ*L of 0.4 mol/L iodoacetic acid and then neutralized in excess NaHCO_3_. After incubation in the dark at room temperature for 1 h, 2 *μ*L of alcoholic 1-fluoro-2,4-dinitrobenzene (1.5/98.5 mL absolute ethanol) was added to each sample and allowed to react in the dark for 8 h. Samples (25 *μ*L injections) were run on a Waters Alliance 2695 system using Varian (Rainin) Microsorb 5-μmol/L amino 25 cm × 4.5 cm columns at room temperature for 35 min with a flow rate of 1 mL/min, with detection at 350 nm.

### Electrical stimulation and muscle contractile characteristics

Isometric diaphragm contractility was assessed as described previously (Tupling et al. 2007). Following the 10-day BSO treatment period, animals were anesthetized with intraperitoneal injection of 0.65 mg/kg pentobarbital sodium. Strips of costal diaphragm were trimmed from the central tendon to ribcage and mounted vertically in a Radnoti-jacketed muscle bath containing oxygenated (95% O_2_, 5% CO_2_) Krebs solution (in mmol/L: 118 NaCl, 25 NaHCO_3_, 11 glucose, 1.2 KHPO_4_, 1.9 CaCl_2_, and 1.2 MgSO_4_; pH 7.4) at 33°C between a plexiglass clamp and a dual mode servomotor (Cambridge Technologies, model 300H Dual Mode Servo) used to measure force. After 30 min of incubation, muscle length (*L*_o_) was adjusted to obtain maximal isometric twitch force. Supramaximal stimulation was applied by a Grass S88 stimulator (Grass Instruments) via closely flanking platinum wire electrodes with pulse duration 0.2 msec. Force data were collected online using a 640-A signal interface (Aurora Scientific) connected to a National Instruments 16-bit analog-to-digital card, and analyzed using Dynamic Muscle Control and Data Acquisition (DMC) and Dynamic Muscle Analysis (DMA) Software (Aurora Scientific).

### Fatigue protocol

To examine the effects of aging and BSO treatment on fatigability of diaphragm muscles, 100 Hz electrical stimulation was applied to the diaphragm strips for 350 msec once per second for 5 min as performed previously (Tupling et al. 2007). Fatigue was assessed as the number of contractions required to reduce maximum force by 50% (50% rundown) and the overall percent reduction in peak force following the 5-min stimulation protocol.

### Normalization of force to cross-sectional area

Following the contractile measurements, the length of the muscle strip was measured and the muscle tissue was separated from the rib and tendon, blotted dry, and weighed. Force data were normalized to the cross-sectional area (CSA) of the strip, which was determined by dividing muscle mass (mg) by the product of *L*_o_ (mm) and the density of mammalian skeletal muscle (1.06 mg/mm^3^) (Mendez and Keys 1960).

### Homogenate preparation

Homogenates were prepared from freshly dissected diaphragm and diaphragm strips following the fatiguing protocol. A total of 20–30 mg of tissue was diluted 11:1 (vol/wt) in ice-cold buffer containing (in mmol/L) 250 sucrose, 5 HEPES, 10 NaN_3_, and 0.2 PMSF, pH 7.5, and homogenized using a hand-held glass homogenizer (Duall 20, Kontes). The homogenates were separated into multiple aliquots and frozen in liquid nitrogen for later analysis. Total protein concentration in homogenates was measured using the method of Lowry, as modified by Schacterle and Pollack (Schacterle and Pollack 1973).

### Ca^2+^-dependent ATPase activity

To examine SERCA function in response to BSO treatment, aging, and fatiguing stimulation, Ca^2+^-dependent ATPase activity was measured using a spectrophotometric plate reader assay at 37°C as described previously (Duhamel et al. 2007). Briefly, muscle homogenates were diluted in reaction buffer containing in mmol/L: 200 KCl, 20 HEPES, pH 7.0, 10 NaN_3_, 1 EGTA, 15 MgCl_2_, 10 phosphoenolpyruvate, 5 ATP, 1 Ca^2+^-ionophore A-23187 (Sigma), as well as 18 U/mL of both lactate dehydrogenase and pyruvate kinase. The mixture was divided in five equal aliquots with pCa ranging from 7.0 to 5.0. Samples were loaded in duplicate into a 96-well plate and the reaction was initiated by the addition of 0.3 mmol/L NADH to each well. ATPase activity was also assessed in the presence of 130 μmol/L cyclopiazonic acid, a highly specific SERCA inhibitor (Goeger et al. 1988; Seidler et al. 1989; Inesi and Sagara 1994), to determine background ATPase activity. Maximal SERCA activity was determined for each sample as the difference between the total ATPase activity of the group of wells with the highest ATPase activity and the background ATPase activity measured with cyclopiazonic acid, and then normalized to total protein concentration.

### Western blotting, immunoprecipitation, and oxidation markers

SERCA1a and SERCA2a content in the costal diaphragm were measured by loading homogenates (SERCA1a: 0.25 *μ*g, SERCA2a 35 *μ*g) into 7.5% polyacrylamide gels and separating proteins using standard SDS-PAGE techniques (Laemmli 1970). Following transfer of the proteins to polyvinyl difluoride membranes (Bio-Rad), the membranes were blocked with 5% skim milk and probed using either anti-SERCA1a monoclonal antibody A52 (Zubrzycka-Gaarn et al. 1984) or anti-SERCA2a antibody 2A7-A1 (Affinity Bioreagents Inc.). Secondary probing was performed using goat-anti-mouse antibody conjugated with horseradish peroxidase (Santa Cruz Biotechnology). Signals were detected using an enhanced chemiluminescence kit (GE Healthcare) using a bioimaging system and densitometric analysis was performed using GeneTools software (Syngene). Quantification of SERCA1a and SERCA2a was performed by generating standard curves (density vs. known amounts of pure SERCA1a or SERCA2a sample prepared as described previously; [Smith et al. 2013]). A known standard sample was included on each gel. This standard sample was used to normalize the optical density of the sample gel to that of the standard curve. The content of SERCA1a or SERCA2a in the samples was then calculated by comparing the adjusted optical densities against the standard curve.

To examine redox modifications to SERCA1a reactive carbonyls, 3-nitrotyrosine and glutathione content were assessed in immunopurified SERCA1a. Immunoprecipitation was performed by centrifugation of samples (200 *μ*g total protein in 500 *μ*L immunoprecipitation buffer [Tris 10 mmol/L, sucrose 300 mmol/L, CHAPS 0.5%, 1 complete protease inhibitor tablet per 50 mL buffer], pH 7.4; Roche) at 6000 *g* for 10 min at 4°C to remove cellular debris. Supernatants were then rotated at 4°C with 1 *μ*g anti-SERCA1a monoclonal antibody A52 (Zubrzycka-Gaarn et al. 1984) for 60 min. Twenty microliters of 50% protein G-sepharose slurry was added to each sample and left rotating at 4°C for 2 h. Samples were washed three times with immunoprecipitation buffer and rotated at 4°C for 10 min between successive washes. Washed beads for glutathione and 3-nitrotyrosine samples were diluted in 2X sample buffer. Washed beads for reactive carbonyl analysis were treated with 10 *μ*L of 5% SDS. Samples were diluted to 10 *μ*g/*μ*L and 6 *μ*L of 10 mmol/L 2,3-dinitrophenylhydrazine in 2 mol/L HCl was added to each sample. Samples were mixed and left for 15 min at room temperature. Samples were neutralized using 6 *μ*L of 2 mol/L Tris in 30% (vol/vol) glycerol and then 9 *μ*L of 2X sample buffer and 15 *μ*L of 4x stacking buffer were added to each sample. SERCA1a content was analyzed for each immunoprecipitated sample using Western blotting techniques and used to normalize the glutathione, 3-nitrotyrosine, and reactive carbonyl values which were probed using antiglutathione (Virogen), antinitrotyrosine (Cayman Chemical Company), and antidinitrophenyl (Sigma) antibodies, respectively. A loading standard was prepared from a sample of homogenate from CTL rats and used to normalize each gel to correct for transfer and exposure differences between gels. Gels used for SERCA1a normalization were transferred onto a single membrane and detected simultaneously, and therefore did not require a loading standard.

### Biotinylated iodoacetamide (BIAM) binding to SERCA1a

BIAM binding to SERCA1a was performed as previously described (Lancel et al. 2009). Muscle samples were diluted in homogenizing buffer (described above) at pH 6.5. Samples were spun at 16,000 *g* for 30 min to precipitate any large cellular debris. The supernatant was sampled for subsequent determination of total SERCA1a content of the sample, while the pellet was discarded. BIAM (0.1 mmol/L final concentration) was added to the remaining supernatant and left to rotate for 2 h at 4°C. Streptavidin sepharose slurry (GE Healthcare) was added to each tube, and left for another 2 h at 4°C to bind to the BIAM. Samples were then spun on a desktop centrifuge to precipitate the sepharose beads and the supernatant was discarded. The beads were then washed with homogenizing buffer (pH 6.5) and spun down (x4) to remove all unbound SERCA. Laemmli buffer was then added to separate BIAM, SERCA1a, and the sepharose beads. The amount of SERCA1a precipitated by this procedure was then compared to the total SERCA1a for each sample using standard Western blotting techniques.

### Statistical analysis

To determine the effects of BSO treatment and aging, two-way factorial ANOVA analysis was performed in conjunction with Tukey’s method of post hoc testing. When the effects of fatigue were also of interest, a three-way mixed effects ANOVA was used with BSO versus CTL and MA versus AD as between-group factors and Basal versus Fatigued as a repeated measure. Significance was taken at *P *<* *0.05. All data are presented as mean ± standard error.

## Results

### General characteristics

Detailed description of food and water intake and changes in body mass during the 10-day treatment period has been published elsewhere (Denniss et al. 2011). Briefly, AD CTL rats maintained a stable body weight throughout the treatment period, whereas the body weight in MA CTL rats was significantly decreased by 2.5 ± 0.5%, despite equivalent food and water intake. BSO treatment resulted in significant declines (*P *<* *0.05) in the body weights of AD and MA BSO groups with reductions of 4.2 ± 0.5% and 8.8 ± 1.3%, respectively, corresponding to significantly lower food intake. To the authors’ knowledge, this is the first quantification of food intake during BSO treatment to be reported, though we have previously reported weight loss in BSO-treated rats (Ford et al. 2006). Based on water consumption, the estimated BSO dosage was 2.5–3.5 mmol/kg/day, not accounting for spillage or water otherwise not consumed by the rats.

### Glutathione measurements

**Figure 1:**
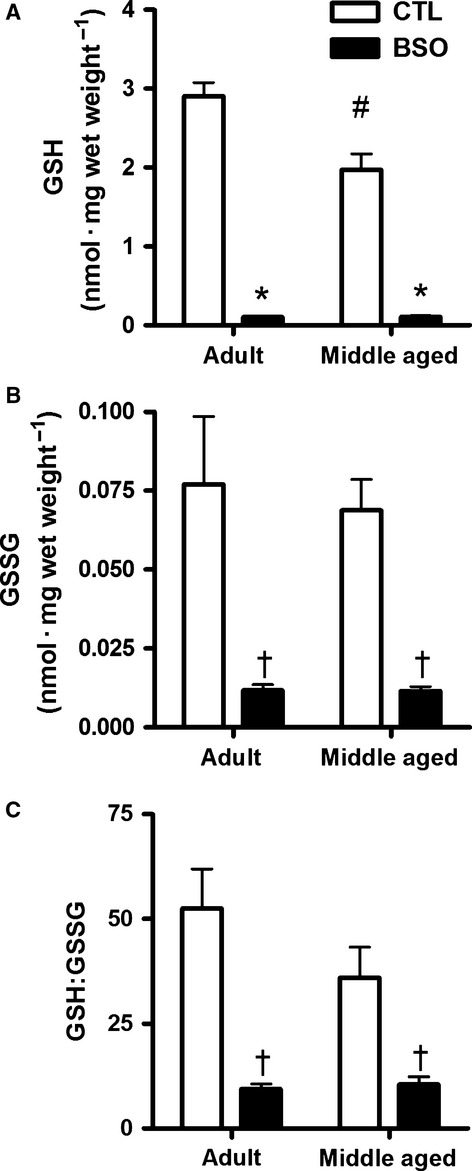
High-performance liquid chromatography was used to measure reduced (A) and oxidized (B) forms of glutathione and the ratio of reduced (GSH) to oxidized (GSSG) glutathione (C) in homogenates prepared from freshly dissected costal diaphragm of control (CTL) and BSO-treated rats. Values are mean ± SEM; adult rats: *n *=* *8 per group, middle aged rats: *n *=* *12 per group. Values were compared using a two-way factorial ANOVA. Interaction effects were found in the total GSH content. *Lower than age-matched control (*P *<* *0.001). ^#^Middle aged CTL < Adult CTL (*P *<* *0.001). ^†^Main effects of BSO treatment were seen in GSSG (CTL > BSO; *P *<* *0.001) and GSH:GSSG ratio (CTL > BSO; *P *<* *0.01). No significant interaction effects were found for either GSSG content or GSH:GSSG ratio.

Examination of GSH content revealed significant (*P *<* *0.05) interaction effects, where BSO treatment resulted in 96% and 95% lower concentrations of GSH (Fig. 1A) relative to the age-matched CTL groups, and AD CTL rats had more GSH than MA CTL rats (*P *<* *0.05). GSSG content was significantly lower in BSO-treated animals (main effect, *P *<* *0.05; Fig. 1B), but there were no age effects in GSSG content. The ratio of GSH:GSSG in BSO-treated rat diaphragm was reduced by 83% and 71% in AD and MA rats, respectively (main effect, *P *<* *0.05; Fig. 1C). The GSH:GSSG ratio in the diaphragm of the MA CTL rats was not statistically different than that of the AD CTL rats.

### Muscle contractility and fatigability

**Figure 2:**
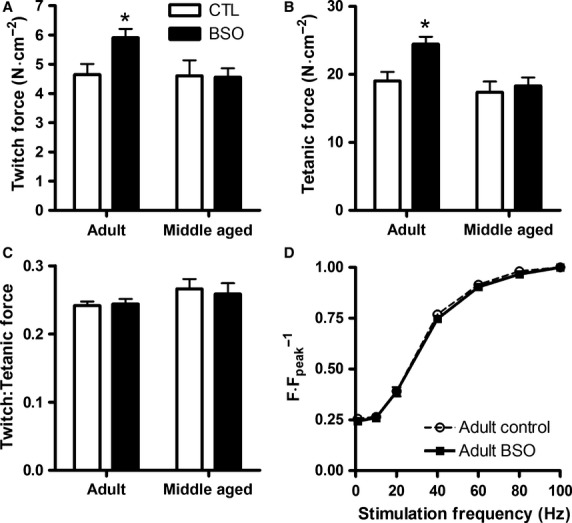
Basal isometric twitch (A) and 100 Hz tetanic force (B) of rat costal diaphragm strips. Adult CTL and middle aged BSO *n *=* *13; middle aged CTL *n *=* *12; adult BSO *n *=* *9. Values are presented as mean ± SEM. *Value is different than all other conditions (*P *<* *0.05). No significant differences were found in the ratio of twitch to tetanic force (C), nor was there a shift in the force–frequency relationship of the adult rats (D; *n *=* *6 per condition).

The effects of BSO treatment on in vitro basal (i.e., unfatigued) peak twitch force and peak tetanic force (100 Hz) are depicted in Figure 2. Twitch and tetanic force were higher in the AD BSO diaphragm than all other groups (interaction effect; *P *<* *0.05), but there were no group differences in the ratio of twitch-to-tetanic force to suggest altered Ca^2+^ sensitivity. The force–frequency relationship was assessed in a subset of AD muscles (*n *=* *6) and no shifts in the relationship were found (Fig. 2D). BSO treatment did not affect twitch kinetics, though the MA groups had longer time to peak tension and contraction times (main effects, *P *<* *0.05; Table 1). The subphysiological temperature used to assess diaphragm function (i.e., 33°C vs. 37°C) would have slowed the contractile kinetics relative to the values seen using an in vivo preparation. Although the force–frequency relationship was not assessed in the MA groups, the slower twitch kinetics of the MA diaphragm than the AD diaphragm would support a lower twitch fusion frequency suggesting that 100 Hz stimulation was sufficient to cause similar if not greater levels of activation in the MA diaphragm than those seen in the AD diaphragm.

**Table 1:** Effects of aging and BSO treatment on costal diaphragm contractility and fatigability

Measure	Adult CTL	Middle aged CTL	Adult BSO	Middle aged BSO
TPT (msec)	26.7 ± 0.5	31.5 ± 1.0*	27.0 ± 0.6	33.2 ± 1.5*
1/2RT (msec)	26.3 ± 0.7	27.6 ± 1.1	27.3 ± 0.7	29.0 ± 0.9
TCT (msec)	53.0 ± 1.2	59.1 ± 1.6*	54.3 ± 1.2	62.2 ± 1.7*
+*df/dt* (N/s/mm)	3.48 ± 0.35	3.91 ± 0.39	3.86 ± 0.48	4.19 ± 0.34
–*df/dt* (N/s/mm)	–1.19 ± 0.13	–1.32 ± 0.14	–1.29 ± 0.17	–1.25 ± 0.11
50% Rundown	56 ± 3	50 ± 3*	52 ± 3†	45 ± 2*†
Final force (% initial)	14.5 ± 1.6	11.6 ± 1.1*	12.9 ± 0.8	10.4 ± 1.0*

Values are mean ± standard error; Adult CTL *n *=* *13, Adult BSO *n *=* *9, Middle Aged CTL *n *=* *14, Middle Aged BSO *n *=* *14. 1/2RT: half relaxation time, measured as the time from peak twitch force to 50% peak twitch force during relaxation phase of twitch. TPT: time to peak tension; measured as time from initial onset of force development to time of peak isometric twitch force. TCT: twitch contraction time; defined as sum of 1/2RT and TPT. +*df/dt*: maximal rate of force development normalized to muscle cross-sectional area. -*df/dt*: maximal rate of relaxation normalized to muscle cross-sectional area. 50% Rundown is the number of contractions required to achieve 50% reduction in force during the fatiguing protocol. Final force refers to the force in the final contraction in the fatiguing protocol as a percentage of the force in the first contraction in the fatiguing protocol.

Main effect of age (Middle aged < Adult; *P *<* *0.05).

Main effect of BSO treatment (Control > BSO; *P *<* *0.05).

Both the time to 50% rundown and the relative force in the final contraction indicated that the MA muscle was more fatigable than the AD muscle (main effects: MA < AD, *P *<* *0.05; Table 1). Although BSO-treated rats had generally lower values for 50% rundown and force in the final contraction, these differences were only significant for the 50% rundown (main effect, *P *<* *0.05). No interaction effects for BSO treatment and age were found.

### SERCA content

**Figure 3:**
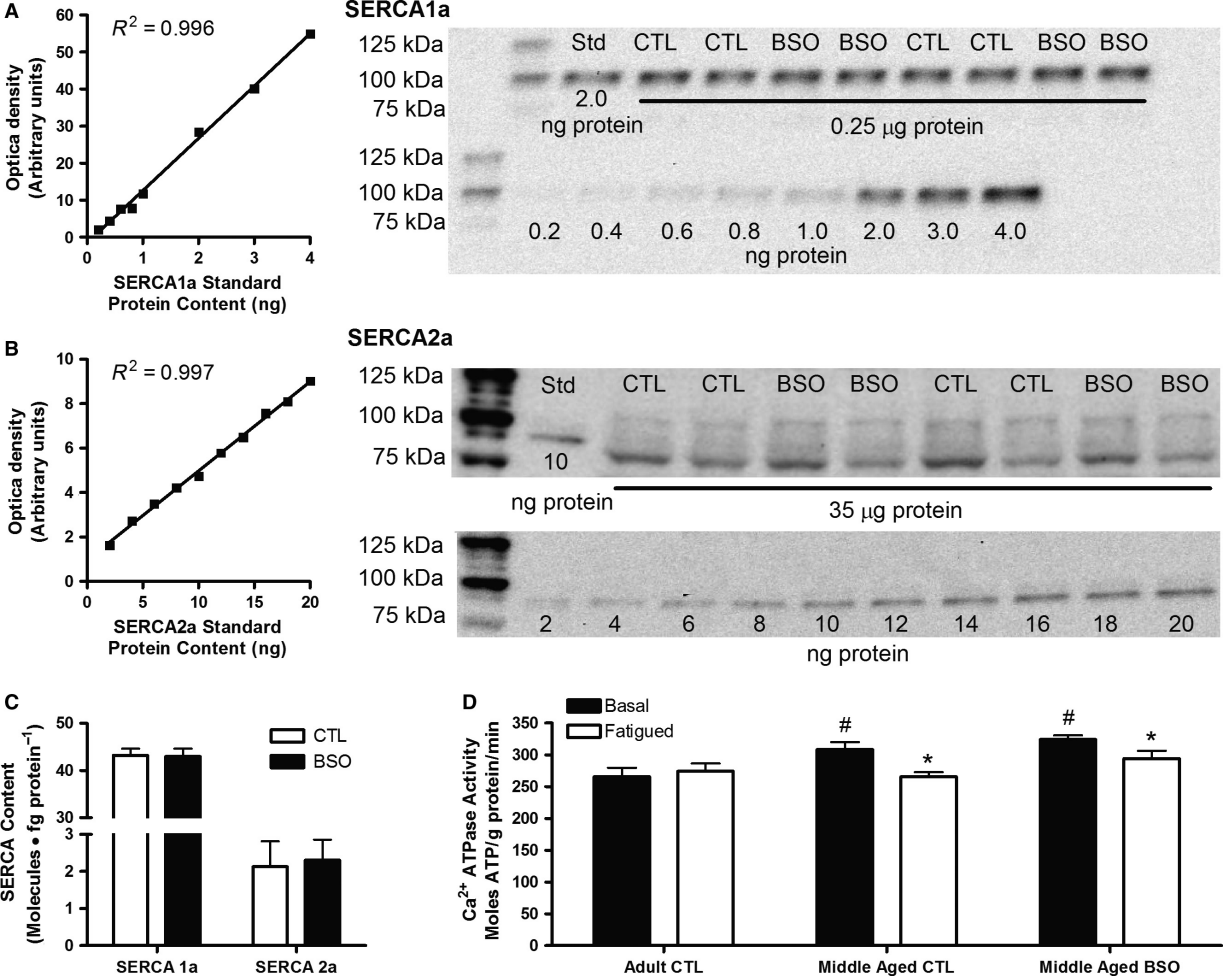
SERCA1a (A) and SERCA2a (B) content were determined for middle aged control (CTL) and BSO-treated rat costal diaphragm using quantitative Western blotting analysis. Summary of data are shown in C (*n *=* *12 per condition). Ca^2+^-dependent ATPase activity was measured in adult and middle aged diaphragm samples taken before and after (D) fatiguing stimulation. Note that Ca^2+^-ATPase data for adult BSO is not included here due to limited tissue availability. ATPase data were analyzed using a two-way mixed design ANOVA (between groups: adult control [*n *=* *7] vs. middle aged control [*n *=* *13] vs. middle aged BSO [*n *=* *13]; repeated measures: basal vs. fatigued). Values are mean ± SEM. *Fatigued value < Basal value of the same group (*P *<* *0.05). ^#^Value > Basal Adult CTL (*P *<* *0.05).

No differences were found in the content of either SERCA1a or SERCA2a between the MA CTL and MA BSO groups (Fig. 3A–C). SERCA1a was ˜20 times more plentiful in the costal diaphragm than SERCA2a. As SERCA1a and SERCA2a expression patterns were previously established for BSO-treated adult rats (Tupling et al. 2007), these measures were not repeated in the present study.

### Ca^2+^-dependent ATPase activity

Maximum Ca^2+^-ATPase activity was assessed in vitro to determine the effects of BSO treatment, aging, and fatiguing stimulation on SERCA function (Fig. 3D). In the basal state, AD CTL diaphragm had lower ATPase activity than the MA CTL and MA BSO (interaction effect, *P *<* *0.05). Following fatigue, only the AD CTL group was able to maintain ATPase function, with the MA BSO and MA CTL groups demonstrating reductions of 9% and 12%, respectively, compared to the associated basal states (interaction effects, *P *<* *0.05). The ATPase activity in the AD BSO group was similar to that seen in the MA groups with a mean basal state activity of 335 ± 7 moles ATP • g protein^–1^ • min^–1^ and declined by 11% in the fatigued state. Due to tissue limitations, only two samples were available for the AD BSO group, thus the AD BSO group was excluded from the statistical analysis of Ca^2+^-ATPase activity.

### SERCA1a oxidation and nitrosylation

**Figure 4:**
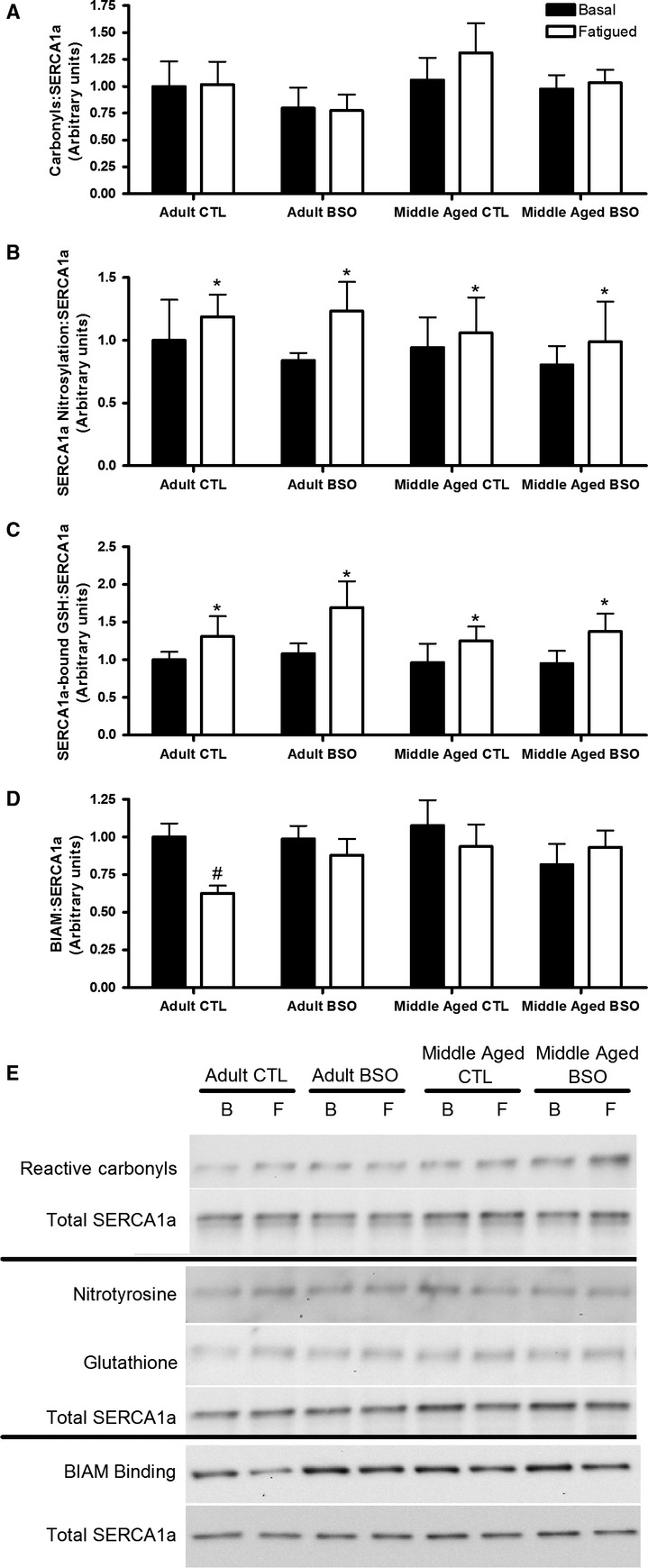
Carbonylation (A), nitrosylation (B) glutathionylation (C), and BIAM binding (D) of immunopurified SERCA1a samples collected before (basal; B) or after (fatigued; F) a fatiguing protocol. Values are mean ± SEM; *n *=* *7 per condition. Representative Western blots are shown in E. All values were normalized to the total SERCA1a content of the purified samples, with nitrotyrosine and glutathione levels measured in the same precipitate. Data were analyzed by three-way mixed effects ANOVA. *Main effect: Fatigued > Basal; *P *<* *0.05. ^#^Interaction effect: Fatigued value < Basal value of the same group; *P *<* *0.05.

Neither BSO treatment nor aging affected the levels of reactive carbonyls, 3-nitrosylation, glutathionylation, or BIAM binding of purified SERCA1a in the basal state (Fig. 4). However, SERCA1a from fatigued samples had significantly higher levels of 3-nitrosylation and glutathionylation than samples taken in the basal state (main effects: Fatigued > Basal, *P *<* *0.05), though carbonylation was not affected by the fatiguing protocol. BIAM binding, an indicator of free cysteine residues, was lower in the fatigued AD CTL group than the basal samples of the AD CTL group (interaction effect: *P *<* *0.05), an effect which was not seen in any other group.

## Discussion

### Redox status

Although we did not directly measure ROS in the diaphragm, we have previously reported that the liver H_2_O_2_ content of the animals used in this study was 46% higher in AD BSO and 80% higher in MA BSO relative to CTL liver at the same ages (Denniss et al. 2011). The declines in GSH:GSSG ratios (Liver: AD 31% and MA 25% vs. Diaphragm: AD 83% and MA 71%) and reductions in GSH content (Liver: AD 42% and MA 60% vs. Diaphragm: AD 96% and MA 95%), were more pronounced in the diaphragm than the liver, suggesting that BSO treatment may have increased ROS/RNS concentrations to an even greater extent in the diaphragm than the liver. Additionally, concurrent analysis of the quadriceps muscles of the MA rats used in the present study revealed that ROS production was 71% higher in the BSO-treated rats than the CTL groups (Dam et al. 2012). Therefore, it is probable that the BSO treatment was successful in causing an oxidative shift in the redox state of the diaphragm. We can also speculate that oxidative stress was averted by upregulating the capacity of other antioxidant systems. For example, the MA BSO-treated quadriceps had 160% higher catalase expression than the MA CTL, at least partially compensating for the loss in H_2_O_2_ scavenging ability caused by GSH depletion (Dam et al. 2012), though the compensatory effects were not universal as the superoxide scavengers, copper–zinc superoxide dismutase and manganese superoxide dismutase were not affected by BSO treatment (Dam et al. 2012).

### Diaphragm contractility

No force differences were seen between the AD and MA animals in the present study. Little information is available on the diaphragm function in middle aged rats, particularly for the Sprague Dawley rats, making it difficult to draw specific comparisons to previous research. However, force declines are commonly seen in the diaphragm of senescent (20+ months of age) F344 rats (e.g., Criswell et al. 1997; McMullen et al. 2011; Cacciani et al. 2014), though this finding is not universal (e.g., Lawler et al. 1997).

Redox modifications are thought to primarily affect muscle contractility by increasing myofibrillar Ca^2+^ sensitivity (Lawler et al. 1997; Andrade et al. 1998, 2001; Lamb and Posterino 2003; Posterino et al. 2003). As both twitch and tetanic forces were elevated in the AD BSO diaphragm relative to AD CTL, and there were no changes in the force–frequency relationship or twitch-to-tetanus ratio between these groups to suggest a change in Ca^2+^ sensitivity or Ca^2+^ availability, force must have been increased by another means. Application of low-dose peroxide has been reported to increase maximal tetanic force (Andrade et al. 2001; Plant et al. 2001). Although the precise mechanism by which peroxide enhances cross-bridge function is not presently clear, a similar mechanism may have caused the elevated tetanic force seen in AD BSO muscle.

As ROS production is temperature dependent (van der Poel et al. 2008), the experimental temperature of 33°C used in the present study may have attenuated basal ROS production relative to in vivo levels. This may have reduced any differences in basal ROS production or accumulation caused by differences in age and limited our ability to detect changes in basal contractile function between the AD and MA groups. However, the lack of force differences between the CTL and BSO-treated MA diaphragm is consistent with the findings of Lawler et al. (Lawler et al. 1997) who report that shifting the redox state of diaphragm bundles with xanthine oxidase increased force in young muscles, but had no effect on the aged muscles at 36°C. As the twitch-to-tetanus ratio was similar between all groups, BSO-induced changes in Ca^2+^-sensitivity of the MA muscle cannot readily explain the age-dependent effects of BSO treatment on force. Therefore, these results are then best explained by age-dependent differences in redox sensitivity of force production.

### Diaphragm fatigability

ROS production has been demonstrated to increase and cause fatigue in isolated diaphragm muscle during repetitive stimulation, even when cellular antioxidant levels are not otherwise impaired (Reid et al. 1992). Studies examining age-dependent differences in ROS/RNS production yield mixed results (e.g., (Nabben et al. 2008; Vasilaki et al. 2006), the possibility that the MA diaphragm produced more ROS than the AD diaphragm during the fatiguing protocol cannot be discounted. The diminished reservoir of antioxidants in the MA and BSO-treated groups may have impaired the ability of these muscles to cope with the increased ROS produced during the fatiguing protocol. Accordingly, the increased number of contractions to reach 50% fatigue seen with BSO treatment and MA in this study may reflect these differences in antioxidant capacity. Although final force was reduced by both BSO treatment and aging, the declines were only significant with aging. Why the number of contractions to 50% fatigue should be significantly reduced with BSO treatment but not the final force may be related to the very high level of fatigue induced by our 5-mi stimulation protocol, diminishing our ability to detect differences between groups in this particular measure. The decreased number of contractions to reach 50% fatigue in MA and BSO-treated diaphragm is consistent with a scenario where the fatiguing stimulation induces greater oxidative stress in MA and BSO-treated animals than AD and CTL animals, culminating with the MA BSO-treated group experiencing the most fatigue. While previous investigations have shown that fatigability of adult rat diaphragm is increased following BSO treatment (Morales et al. 1994; Tupling et al. 2007), our results extend this statement to include middle aged rat diaphragm.

### SERCA isoforms, activity, and oxidation state

We have previously reported that maximal Ca^2+^-ATPase activity and maximal Ca^2+^-uptake are lower in adult diaphragm than adult BSO-treated diaphragm (Tupling et al. 2007). Here, we extend these findings to demonstrate that maximal Ca^2+^-ATPase activity in unfatigued diaphragm was lower in AD CTL than MA BSO and MA CTL diaphragm, and cautiously (due to the low n value for Ca^2+^ATPase activity in BSO-treated AD diaphragm), lower than AD BSO as well. SERCA1a expression has generally been found to be stable to changes in oxidative conditions (Ferrington et al. 1998; Tupling et al. 2007; Thomas et al. 2010) with few exceptions (Malyshev et al. 2000). Accordingly, no changes in SERCA1a expression were seen in this study or our previous assessment (Tupling et al. 2007). In contrast, SERCA2a expression has been shown to increase in situations associated with elevated exposure to ROS, including administration of nitric oxide donors (Malyshev et al. 2000), exercise (Thomas et al. 2010), aging (Thomas et al. 2010), and BSO treatment (Tupling et al. 2007). As previously reported (Tupling et al. 2007), increases in SERCA2a expression in adult BSO diaphragm can at least partially account for the increased activity seen in AD BSO relative to AD CTL. BSO treatment did not cause SERCA2a expression to increase in MA rats. As SERCA expression was not directly compared between AD and MA rats, it remains possible that the diaphragm of MA rats express more total SERCA than that of the AD rats, particularly in the absence of differences in basal carbonylation, nitrosylation, glutathionylation, or BIAM binding in purified SERCA1a between any groups. Similar measures were not performed on immunopurified SERCA2a as its contribution to total Ca^2+^-ATPase would be small since the rate of SERCA1a ATPase activity is approximately twice that of SERCA2a (Sumbilla et al. 1999), and SERCA2a accounted for only 5% of the total SERCA in the MA diaphragm, a value comparable to the 10% contribution reported by Wu and Lytton (Wu and Lytton 1993).

In samples collected following the fatiguing protocol, maximal Ca^2+^-ATPase activity was lower than in the basal state in both MA groups, and cautiously, in the AD BSO group as well (low *n* value). However, Ca^2+^-ATPase activity was maintained in the AD CTL group. Fatiguing stimulation increased SERCA1a nitrosylation and glutathionylation in all groups. Interestingly, BIAM binding to SERCA1a was decreased by the fatiguing stimulation exclusively in the AD Control group. BIAM has been demonstrated to specifically bind to reduced cysteine-674 in SERCA2a isoforms (Lancel et al. 2009). Reversible S-glutathionylation of cysteine-674, which enhances the pump activity of SERCA2a (Adachi et al. 2004), will prevent BIAM binding. Given the large degree of functional and sequence homology between SERCA1a and SERCA2a, there is a high likelihood that BIAM will also specifically bind to cysteine-674 in SERCA1a, and that cysteine-674 is an important regulatory site in SERCA1a. Should this specificity of BIAM binding hold true for multiple SERCA isoforms, it can be speculated that cysteine-674 of SERCA1a can also be S-glutathionylated during stimulation and enhance the functional capabilities of the enzyme. The reduction in BIAM binding following fatigue in only the AD CTL group may therefore account for the maintenance of Ca^2+^-ATPase activity in the AD CTL group following fatigue. Future work is required to determine the specific interactions between BIAM and SERCA1a.

### Summary

This study aimed to determine how BSO treatment affects the contractility and SERCA function of middle aged and adult rat diaphragm. Drawing on the results of the present and our previous study (Tupling et al. 2007), in adult diaphragm, GSH depletion by BSO treatment causes specific force, basal Ca^2+^-ATPase activity, SERCA2a expression, and fatigability to increase. In contrast, treatment of middle aged rats with BSO only resulted in an increase in fatigability. Both BSO treatment and middle age status were associated with an inability to decrease BIAM binding to SERCA1a following fatigue, suggesting a loss of oxidative control over the enzyme. Collectively, the results of this study demonstrate the importance of redox signaling in the functional control of contractile activity and SERCA function, and how this control appears to be lost with time, becoming apparent by middle age. The insights gained from this study advance our understanding of both the oxidative changes that occur with age and their functional consequences.
